# Supplementary material for: A Functional Chemiluminescent Probe for in Vivo Imaging of Natural Killer Cell Activity Against Tumours
Source: Angew Chem Weinheim Bergstr Ger. 2021 Feb 2;133(11):5763–7. doi: 10.1002/ange.202011429 (PMC10946790; doi:10.1002/ange.202011429)
Supplement: Supplementary file 1 — Supplementary [file ANGE-133-5763-s001.pdf]

Angewandte Chemie
Eine Zeitschrift der Gesellschaft Deutscher Chemiker

Supporting Information

A Functional Chemiluminescent Probe for in Vivo Imaging of Natural Killer Cell Activity Against Tumours

*Jamie I. Scott, Sara Gutkin, Ori Green, Emily J. Thompson, Takanori Kitamura,**
Doron Shabat, and Marc Vendrell**

ange_202011429_sm_miscellaneous_information.pdf

Supplementary Information

A Functional Chemiluminescent Probe for *in vivo* Imaging of Natural Killer Cell Activity against Tumours

Table of Contents

Experimental Details

Supplementary Figures

NMR spectra

Supplementary References

Experimental Details

Materials and methods.

Chemicals and solvents were either A.R. grade or purified by standard techniques. Thin layer chromatography (TLC): silica gel plates Merck 60 F254: compounds were visualised by irradiation with UV light. Column chromatography (FC): silica gel Merck 60 (particle size 0.040-0.063 mm), eluent given in parentheses. Fmoc-amino acids were obtained from Iris Biotech GmbH (Fmoc-Ile-OH, Fmoc-Pro-OH)] and Sigma-Aldrich [Fmoc-Asp(OAll)-OH, Fmoc-Glu(OAll)-OH]. Polystyrene resin with a 2-chlorotrityl chloride linker was obtained from Iris Biotech GmbH. All reagents were used without further purification unless otherwise stated. Spectroscopic data was measured on a Synergy HT spectrophotometer (Biotek) and the data analysis was performed using GraphPad Prism 5.0. Peptide synthesis reactions and products were monitored by HPLC-MS at 220 nm using a HPLC Waters Alliance 2695 with YMC-Pack ODS-AQ, 50 × 4.6 mm, S-3 μm column and a diode array detector. Eluents: H_2O (0.1% HCOOH) and ACN (0.1% HCOOH). Flow: 1.0 mL min^{-1}. The MS detector was configured with an electrospray ionisation source (Micromass ZQ4000) and nitrogen was used as the nebuliser gas. Data acquisition was performed with MassLynx software. ^{1}H-NMR spectra were recorded using Bruker Avance operated at 400 MHz. ^{13}C-NMR spectra were recorded using Bruker Avance operated at 100 MHz. Chemical shifts were reported in ppm on the δ scale relative to a residual solvent ($CDCl_3$: δ = 7.26 for ^{1}H-NMR and 77.16 for ^{13}C-NMR, DMSO-d_6: δ = 2.50 for ^{1}H-NMR and 39.52 for ^{13}C-NMR). Multiplicities are referred by the following abbreviations: s = singlet, d = doublet, t = triplet, dd = doublet doublets, ddd = double double doublet, dt = double triplet, q = quartet and m = multiplet.

DMEM, αMEM and fetal bovine serum were purchased from ThermoFisher Scientific. Human recombinant GZM A and B was purchased from Enzo Life Sciences, human recombinant IL-2 was purchased from Peprotech and human neutrophil elastase was received from Prof. Kev Dhaliwal (University of Edinburgh). Ac-IEPD-AMC was purchased from Enzo Life sciences, Ac-IEPD-CHO was obtained from Abcam. Arginine, glutamic acid, glutathione and hydrogen peroxide were all obtained from Sigma-Aldrich. Work with serum from human peripheral blood complied with all relevant ethical regulations and informed consent was obtained. The study protocol was approved by the Accredited Medical Regional Ethics Committee (AMREC, reference number 20-HV-069) at the University of Edinburgh.

Synthesis and characterisation

Abbreviations. ACN: acetonitrile, DCM: dichloromethane, DIPEA: *N,N*-diisopropylethylamine, DMF: *N,N'*-dimethylformamide, THF: tetrahydrofuran, DMBA: 1,3-dimethylbarbituric acid, EEDQ: *N*-ethoxycarbonyl-2-ethoxy-1,2-dihydroquinoline, COMU: (1-cyano-2-ethoxy-2-oxoethylidenaminooxy)dimethylaminomorpholinocarbenium hexa-fluorophosphate, HBTU: hexafluoro phosphate benzotriazole tetramethyl uronium, Oxyma: ethyl(hydroxyimino)cyanoacetate, Hex: hexanes, TFA: trifluoroacetic acid, TMSCl: trimethylsilyl chloride.

Resin loading. Fmoc-AA-OH (1.4 eq) was attached to the resin with DIPEA (3 eq.) in DCM at r.t. for 10 min and then DIPEA (7 eq) for 40 min. The remaining 2-chlorotrityl groups were capped by addition of adding MeOH (0.8 mL g^{-1} resin) for 10 min. After that, the resin was filtered and washed with DCM (5 × 1 min), DMF (5 × 1 min). The loading of the resin was determined by titration of the Fmoc group using UV-Vis absorbance.

Amino acid coupling. After the Fmoc group was removed, the resin was washed with DMF (5 × 1 min), DCM (5 × 1 min), DMF (5 × 1 min). A solution of the appropriate Fmoc-AA-OH (4 eq), COMU (4 eq), Oxyma (4 eq) and DIPEA (8 eq.) was stirred for 1 min in DMF before addition to the resin previously swollen in DMF. The mixture was then stirred for 1.5 h at r.t. The solution was filtered off and the resin was washed with DMF (5 × 1 min) and DCM (5 × 1 min). The completion of the coupling was monitored by the Kaiser test (or chloranil test when secondary amines involved).

***N*-terminal acetylation.** After removal of the last Fmoc group, the resin was washed with DMF (5 × 1 min), DCM (5 × 1 min), DMF (5 × 1 min) and then treated with a cocktail of

5% Ac_2O and 6% DIPEA in DMF. After that, the resin was filtered off and washed with DCM (5 × 1 min), DMF (5 × 1 min)

Cleavage and purification. The resin-bound peptide was treated with 5% TFA in DCM (5 × 1 min) and washed with DCM. The combined filtered mixtures were poured over DCM and evaporated under reduced pressure. The peptide was purified by precipitation in ice-cold Et_2O generating a white solid (HPLC purity (220 nm) > 95%).

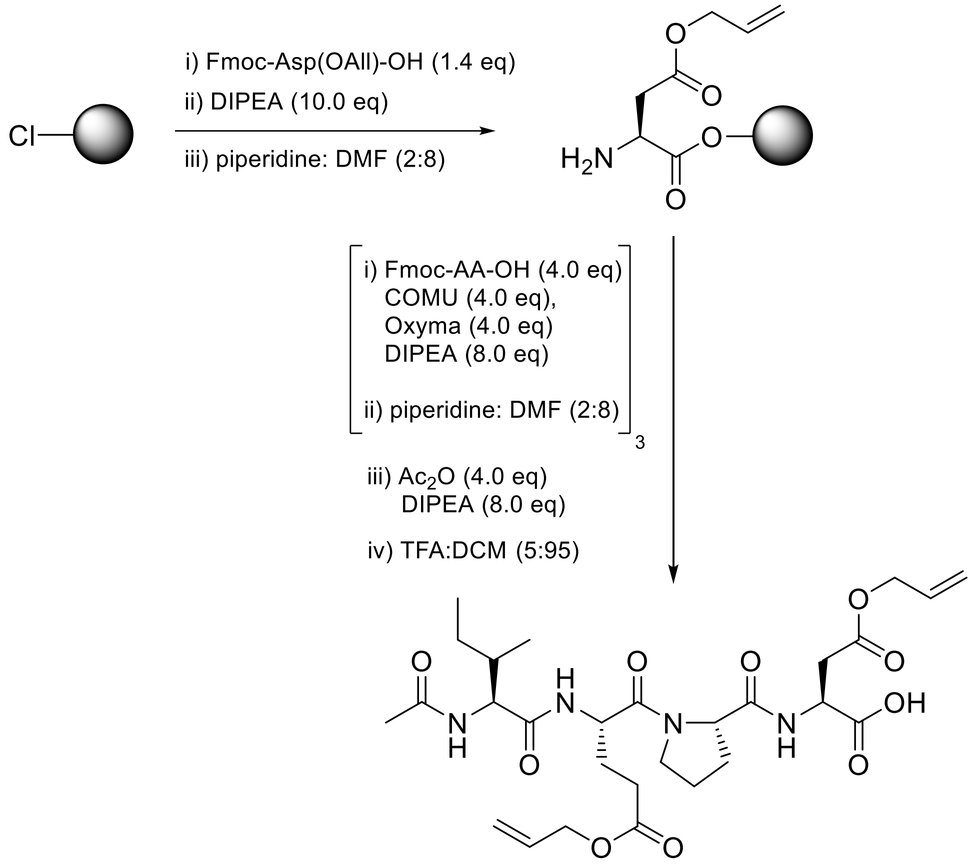

Scheme S1. Solid-phase peptide synthesis of Ac-IE(OAll)PD(OAll)-OH.

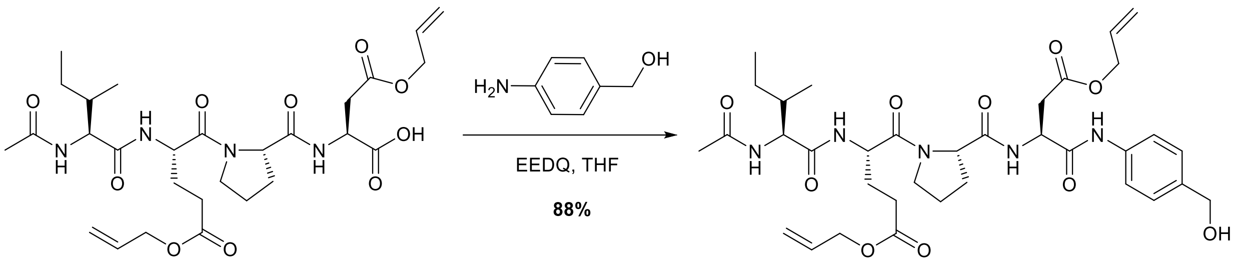

Ac-IE(OAll)PD(OAll)-OH **2**

Compound 2. Aminobenzyl alcohol (24 mg, 0.19 mmol), *N*-ethoxycarbonyl-2-ethoxy-1,2-dihydroquinoline (EEDQ, 67 mg, 0.27 mmol) and Ac-IE(OAll)PD(OAll)-OH (100 mg, 0.16 mmol) were dissolved in 5 ml of THF. The reaction was allowed to warm up to r.t. and monitored by TLC (Hex:EtOAc, 40:60). Upon completion, the reaction mixture was diluted with EtOAc (100 mL) and washed with a solution of HCl 1M (50 mL) and brine (50 mL). The organic layer was separated, dried over Na_2SO_4, filtered and the solvent was evaporated under reduced pressure. The product was purified using column chromatography on silica gel (Hex:EtOAc, 40:60) to afford compound **2** (98 mg, 88% yield) as a white solid.

^{1}H NMR (400 MHz, $CDCl_3$) δ: 8.78 (s, 1H), 7.64 (d, *J* = 8.5 Hz, 1H), 7.62 (dd, *J* = 8.5 Hz, 2H), 7.38 (d, *J* = 7.8 Hz, 1H), 7.34 – 7.23 (d, *J* = 8.4 Hz, 2H), 6.53 (d, *J* = 8.9 Hz, 1H), 5.87 (dd, 2H), 5.38 – 5.14 (m, 4H), 4.91 (dd, 1H), 4.84 (d, 1H), 4.60 (s, 2H), 4.57 – 4.48 (m, 4H), 4.38 (dd, 1H), 3.80 (t, 2H), 3.09 (dd, *J* = 17.1 Hz, 5.5 Hz, 1H), 2.84 (dd, *J* = 17.1 Hz, 5.7 Hz, 1H), 2.36 (m, 2H), 2.29 – 2.12 (m, 3H), 2.10 – 2.00 (m, 4H), 1.75 (dd, 2H), 1.50 (dd, 1H), 1.28 (s,3H), 1.12 (dd, 1H), 0.98 – 0.77 (m, 6H).

^{13}C NMR (100 MHz, $CDCl_3$) δ: 137.4, 137.3, 132.1, 131.8, 127.8, 120.3, 118.8, 118.5, 65.9, 65.6, 64.8, 61.4, 57.7, 50.3, 50.2, 47.9, 37.8, 35.2, 29.8, 29.4, 29.3, 27.1, 25.3, 24.9, 23.3, 15.7, 11.4.

MS (ESI+): m/z calc. for $C_{35}H_{49}N_5O_{10}$: 699.3; found: 700.7 $[M+H]^+$.

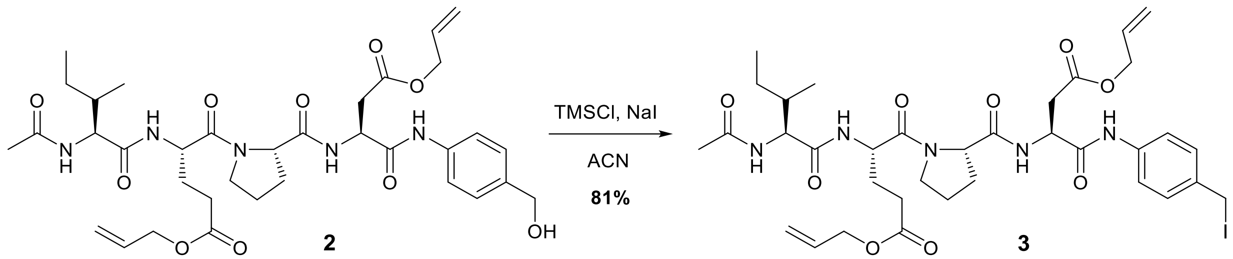

Compound 3. Compound **2** (95 mg, 0.14 mmol) was dissolved in 5 mL ACN and cooled to 0 ºC. Then, sodium iodide (61 mg, 0.41 mmol) was added followed by the rapid addition of TMSCl (52 µL, 0.41 mmol). The reaction was allowed to warm up to r.t. and monitored by TLC (Hex:EtOAc, 60:40). Upon completion, the reaction mixture was diluted with EtOAc, and washed with saturated $Na_2S_2O_3$ followed by brine. The organic layer was separated, dried over Na_2SO_4, filtered and the solvent was evaporated under reduced pressure. The product was purified using column chromatography on silica gel (Hex:EtOAc 60:40) to afford compound 3 (90 mg, 81% yield) as an off-white solid.

^{1}H NMR (400 MHz, $CDCl_3$) δ: 8.83 (s, 1H), 7.64 (d, *J* = 8.5 Hz ,1H), 7.62 (dd, *J* = 8.5 Hz 2H), 7.38 (d, *J* = 7.6 Hz, 1H), 7.34 – 7.23 (d, *J* = 8.5 Hz, 2H), 6.33 (d, *J* = 8.8 Hz, 1H), 5.87 (dd, 2H), 5.38 – 5.14 (m, 4H), 4.91 (dd, 1H), 4.84 (d, 1H), 4.43 (s, 2H), 4.42 – 4.38 (m, 4H), 4.38 (dd, 1H), 3.80 (t, 2H), 3.09 (dd, *J* = 17.1, 5.3 Hz, 1H), 2.84 (dd, *J* = 17.1, 5.9 Hz, 1H), 2.36 (m, 2H), 2.29 – 2.12 (m, 3H), 2.10 – 2.00 (m, 4H), 1.75 (dd, 2H), 1.50 (dd, 1H), 1.28 (s, 3H), 1.12 (dd, 1H), 0.98 – 0.77 (m, 6H).

^{13}C NMR (100 MHz, $CDCl_3$) δ: 173.0, 171.9, 171.7, 170.5, 168.4, 137.7, 135.2, 132.1, 131.7, 129.5, 120.4, 118.8, 118.5, 65.9, 65.6, 61.2, 60.5, 57.7, 50.5, 50.2, 47.9, 37.8, 35.2, 32.0, 31.8, 29.8, 29.6, 29.5, 29.2, 27.0, 25.3, 25.0, 23.4, 22.8, 21.2, 15.7, 14.3, 11.5, 6.0.

MS (ESI+): m/z calc. for $C_{35}H_{49}IN_5O_{10}$: 809.3; found: 810.7 $[M+H]^+$.

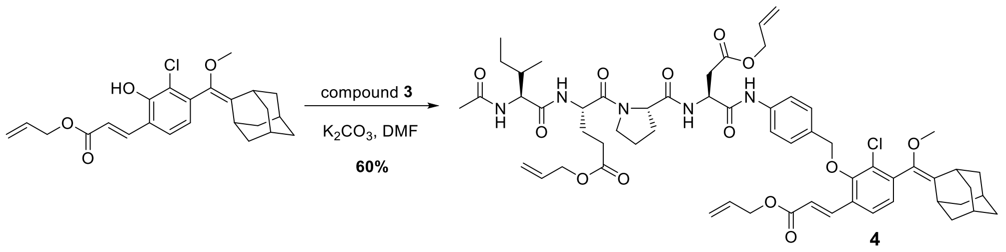

Compound 4. Compound **3** (90 mg, 0.11 mmol) was added to a solution of allyl protected phenol enol ether[1] (50 mg, 0.12 mmol) and K_2CO_3 (23 mg, 0.17 mmol) in DMF (1 mL). The reaction was stirred at r.t. for 1 h and monitored by TLC (Hex:EtOAc, 70:30). Upon completion, the mixture was diluted with EtOAc (100 mL) and washed with 0.5M HCl (50 mL). The organic layer was separated, washed with brine, dried over Na_2SO_4, evaporated under reduced pressure and the crude was purified by column chromatography on silica gel (Hex:EtOAc, 70:30) affording compound **4** as a pale yellow solid (73 mg, 60% yield).

^{1}H NMR (400 MHz, $CDCl_3$) δ: 8.82 (s, 1H), 7.96 (d, *J* = 16.2 Hz, 1H), 7.69 (d, *J* = 8.5 Hz, 2H), 7.64 (d, *J* = 8.6 Hz, 1H), 7.45 (d, *J* = 8.5 Hz, 2H), 7.36 – 7.30 (m, 1H), 7.08 (d, *J* = 8.0 Hz, 1H), 6.49 (d, 1H), 6.35 (d, *J* = 8.7 Hz, 1H), 6.05 – 5.94 (m, 1H), 5.94 – 5.81 (tdd, *J* = 11.0, 7.6, 5.5 Hz, 2H), 5.43 – 5.18 (dddd, *J* = 20.2, 13.9, 2.8, 1.4 Hz, 6H), 5.01 – 4.92 (m, 3H), 4.85 (m, 1H), 4.78 – 4.67 (dd, *J* = 5.7, 1.2 Hz, 2H), 4.64 – 4.52 (ddd, *J* = 5.7, 5.0, 2.8 Hz, 7H), 4.48 – 4.37 (dd, *J* = 8.5, 6.8 Hz, 1H), 3.85 (m, 2H), 3.33 (s, 3H), 3.26 (s, 1H), 3.17 – 3.06 (m, 1H), 2.81 (dd, *J* = 17.1, 5.8 Hz, 1H), 2.52 – 2.40 (m, 2H), 2.24 (m, 4H), 2.11 – 1.67 (m, 18H), 1.54 – 1.45 (m, 2H), 0.97 – 0.79 (m, 6H).

^{13}C NMR (100 MHz, $CDCl_3$) δ: 173.0, 171.9, 171.9, 171.7, 170.5, 168.4, 166.5, 153.9, 139.5, 139.2, 138.4, 138.4, 132.5, 132.3, 132.1, 131.9, 129.7, 125.2, 120.1, 120.0, 118.8, 118.5, 75.9, 65.9, 65.6, 65.5, 61.2, 57.7, 57.4, 50.4, 50.1, 47.9, 39.3, 39.2, 38.7, 37.8, 37.2, 35.2, 33.1, 29.8, 29.6, 29.4, 29.2, 28.5, 28.3, 27.1, 25.3, 24.9, 23.3, 14.2, 11.5.

MS (ESI+): m/z calc. for $C_{59}H_{74}ClN_5O_{13}$: 1095.5; found: 1097.0 $[M+H]^+$.

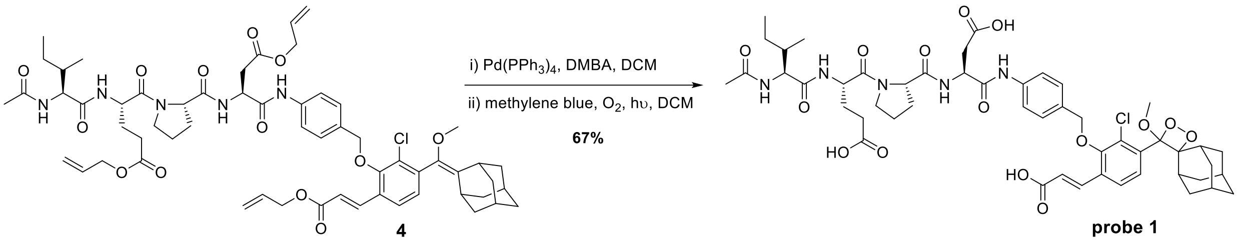

Probe 1. Compound **4** (70 mg, 0.06 mmol) was dissolved in DCM (2 mL), followed by the addition of DMBA (60 mg, 0.38 mmol) and $Pd(PPh_3)_4$ (7 mg, 0.006 mmol). The reaction was stirred at r.t. and monitored by RP-HPLC. Upon complete removal of allyl groups, DCM (10 mL) and a catalytic amount of methylene blue were added. Then, oxygen was bubbled through the solution while irradiating with yellow light. The reaction was monitored by RP-HPLC. Upon completion (~20 min), the solvent was concentrated under reduced pressure and the product was purified by preparative HPLC.

^{1}H NMR (400 MHz, DMSO) δ: 9.71 (s, 1H), 8.25 (d, *J* = 5.3 Hz, 1H), 8.13 (d, *J* = 7.1 Hz, 1H), 7.92 (d, *J* = 8.3 Hz, 1H), 7.88 – 7.80 (d, *J* = 8.9 Hz, 1H), 7.80 – 7.71 (m, 2H), 7.69 (d, *J* = 8.5 Hz, 2H), 7.38 (d, *J* = 8.5 Hz, 2H), 6.64 (d, *J* = 12.6 Hz, 1H), 4.94 – 4.78 (m, 2H), 4.60 (dt, 1H), 4.49 (dd, 1H), 4.25 (dd, *J* = 8.2, 4.8 Hz, 1H), 4.17 (m, 2H), 3.73 – 3.56 (m, 4H), 3.10 (s, 3H), 2.87 (s, 1H), 2.78 (dd, *J* = 16.4, 5.8 Hz, 1H), 2.66 – 2.54 (m, 1H), 2.36 – 2.16 (m, 4H), 1.91-1.81 (m, 5H), 1.71– 1.49 (m, 12H), 1.45 – 1.23 (m, 4H), 1.04 (dd, 1H), 0.85 – 0.72 (m, 6H).

^{13}C NMR (100 MHz, DMSO) δ 174.7, 172.3, 171.9, 170.9, 169.9, 169.7, 167.7, 154.2, 139.7, 137.3, 134.7, 131.9, 131.1, 129.9, 129.0, 127.4, 126.7, 123.6, 119.7, 111.8, 96.0, 76.1, 60.5, 57.1, 51.2, 50.4, 50.0, 47.5, 37.1, 36.3, 33.7, 32.3, 31.7, 31.5, 30.1, 29.6, 26.7, 26.1, 25.8, 25.1, 25.0, 23.1, 15.9, 11.5.

MS (ESI+): m/z calc. for $C_{50}H_{62}ClN_5O_{15}$: 1007.3; found: 1008.9 $[M+H]^+$.

***In vitro* characterisation assays.** Enzymatic assays were run in a 384-well format in a PCR opaque microplate (Thermo Scientific). All experiments were performed at least in triplicate. Dilutions and reactions using human GZM B and human neutrophil elastase were prepared in 50 mM Tris-HCl and 100 mM NaCl (pH 7.4). GZM A assays were performed in 25 mM HEPES, 0.1% CHAPS and 10 mM DTT (pH 7.5). Dilutions and incubations with CASP8 and CASP9 were prepared in 50 mM HEPES, 100 mM NaCl, 0.1% Triton X-100, 10 mM DTT, 1 mM EDTA and 10% glycerol (pH 7.4). Any other tested biomolecules were prepared in PBS. Proteolytic activity was determined by calculating the fold change in chemiluminescence signal (or fluorescence signal in the case of Ac-IEPD-AMC) over background signal provided by the corresponding dilution of the probes with the enzyme using a spectrophotometer (Synergy H1 Hybrid Reader, BioTek) at 37°C. Unless otherwise stated, probes were utilized at a concentration of 100 µM. Enzyme concentration used: 20 nM (except CASP9, which was used at 200 nM) and all other biomolecules were tested at 100 µM. In the case where an inhibitor was used, the enzyme was pre-incubated for 1 h with the inhibitor (100 µM) before addition of probe **1**.

Cell culture. MDA-MB-231 (human breast adenocarcinoma) cells were obtained from ATCC and grown in DMEM supplemented with 10% FBS, antibiotics (100 U mL^{-1} penicillin and 100 mg mL^{-1} streptomycin) and 2 mM L-glutamine in a humidified atmosphere at 37ºC with 5% CO_2. Cells were regularly passaged in T-75 cell culture flasks upon reaching 90% confluency. NK-92 (human natural killer) cells were grown in NK cell media [αMEM media supplemented with horse serum (12.5%), FBS (12.5%), L-glutamine (2

mM), folic acid (20 μM), inositol (200 μM) and 2-mercaptoethanol (100 μM)] in a humidified atmosphere at 37°C with 5% CO_2.

***In vitro* fluorescence imaging.** MDA-MB-231 cells were passaged and resuspended in NK cell media (5×10^5 cells mL^{-1}) and incubated with MitoTracker Red CMXRos (300 nM) for 20 min at 37°C in a humidified atmosphere with 5% CO_2. MDA-MB-231 cells were then washed with NK cell media, pelleted and re-suspended in NK cell media before seeding on a 8-well glass bottom confocal chamber (2,000 cells per well) which had been pre-treated with Geltrex (100 μL per well) for 1 h at 37°C and then aspirated prior to cell addition. NK-92 cells were then added to each well (16,000 cells per well) alongside IL-2 (2,000 U mL^{-1}) and NucView®488 (BioTium) (10 μM) before incubation for 24 h at 37°C in a humidified atmosphere with 5% CO_2. Following 24 h incubation, the co-culture was imaged under EVOS FL Auto 2 imaging system using a 20X objective. NucView was imaged using a GFP filter and MitoTracker Red was imaged using an RFP filter. Images were analysed using ImageJ.

***In vitro* chemiluminescence imaging.** MDA-MB-231 cells and NK-92 cells were co-cultured as described above in white round-bottom 96-well plates (2,000 MDA-MB-231 and 16,000 NK-92 cells per well) at 37°C in a humidified atmosphere with 5% CO_2. Following incubation, probe **1** was added at the indicated concentrations and plates were immediately imaged under a PhotonImager™ (BioSpace Lab) imaging system for a maximum of 15 min and images were processed with M3Vision™ software. For the

inhibitor experiments, Ac-IEPD-CHO was added to the co-cultures at the indicated concentrations 1 h prior to addition of probe **1** (500 μM).

Immunohistochemistry. NK-92 and MDA-MB-231 cells were co-cultured at a 8:1 ratio in NK cell media including IL-2 (2,000 U mL^{-1}). MDA-MB-231 cells were cultured alone as a control. After co-cultures, NK-92 cells were removed by washing with PBS and MDA-MB-231 cells were detached and fixed with 4% PFA. Antigen retrieval was performed with 10 mM sodium citrate before permeabilization with 0.1% Triton X-100 in PBS. Slides were blocked for 1 h at r.t. in 5% (v/v) donkey serum with 0.1% Tween-20 in PBS. Incubation with a rat monoclonal antibody against human GZM B (1:100 dilution) was performed overnight in the dark at 4°C. Then, the secondary antibody Alexa Fluor 647 donkey anti-rat (1: 500 dilution) was incubated for 1 h at r.t. Slides were incubated with Hoechst 33342 before mounting with coverslips, images were acquired on a confocal fluorescence microscope Leica SP8 (λ_{exc}: 405 nm and 633 nm) and processed with ImageJ.

***In vivo* imaging.** Animal testing and research complied with all relevant ethical regulations. The study protocol was approved by the UK Home Office (P526C60B3) and by the University of Edinburgh animal welfare Committee. Mice were housed in a specific-pathogen-free facility with standard husbandry. NOD/SCID gamma (NSG; NOD.Cg-PrkdcscidIl2rgtm1Wjl/SzJ) mice were obtained from Charles River. 1×10^6 MDA-MB-231 human breast cancer cells in 50 μL PBS were mixed with 50 μL growth factor reduced basement membrane (Matrigel, Corning) and subcutaneously injected into both the left and right flanks of female NSG mice aged 8 weeks. After 21 days, 2×10^6 NK-92 cells in

50 μL PBS were injected into only one tumour per mouse. The same volume of PBS alone was injected into the other tumour. After 8 h, 125 μg of probe **1** in PBS were injected into both tumors and mice were imaged immediately after under a PhotonImager™ (BioSpace Lab) imaging system for 15 min and images were processed with M3Vision™ software.

Supplementary Figures

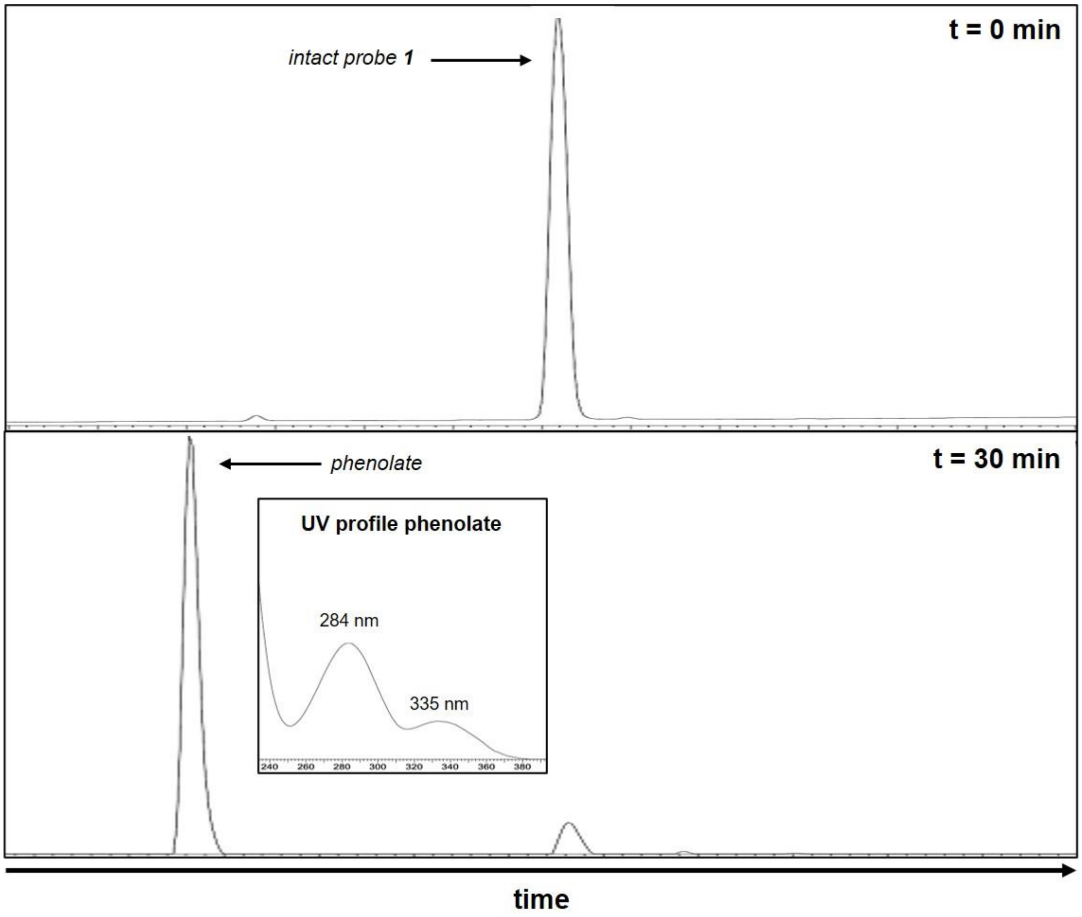

Supplementary Figure 1. HPLC traces of probe 1 before and after reaction with GZM B. HPLC chromatogram (UV detection: 284 nm) of probe **1** (100 μM) before and after reaction (30 min) with recombinant GZM B (20 nM) at 37 ˚C.

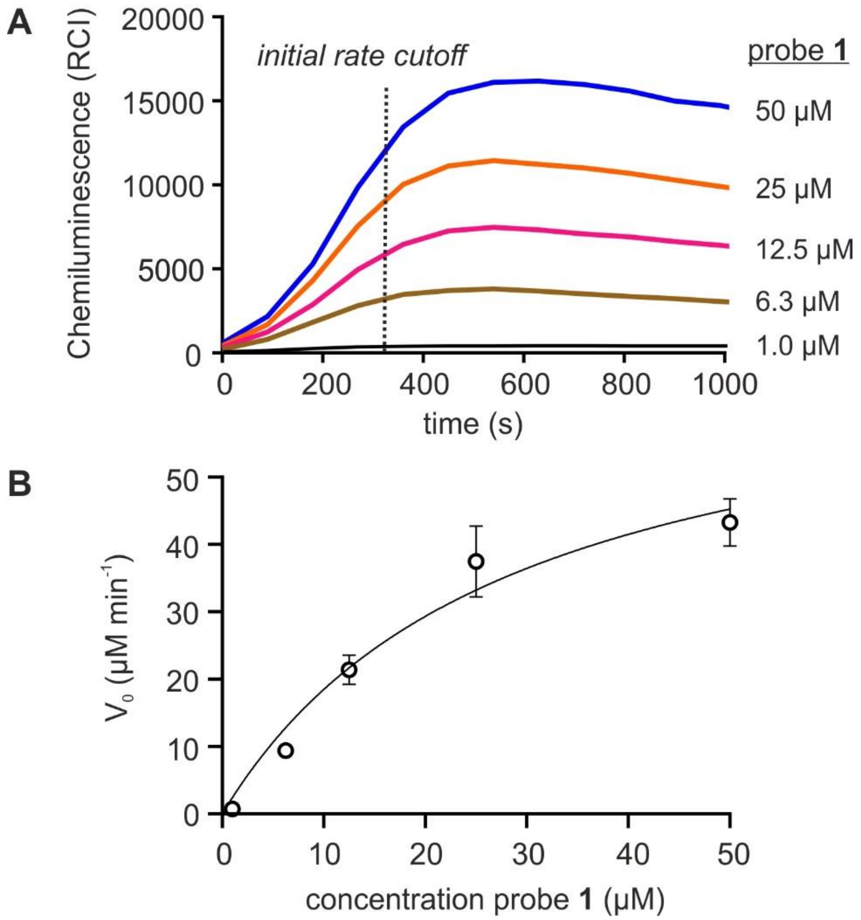

Supplementary Figure 2. Kinetics of probe 1 upon reaction with GZM B. A) Representative (from 3 independent experiments) time-lapse chemiluminescence signals derived from the reaction of hr GZMB (20 nM) and probe **1** at different concentrations. The cutoff indicates the data range used to calculate the V_o for the determination of kinetic constants. B) Cleavage rate of probe **1** plotted against probe concentration. Data presented as means ± SEM with the Michaelis-Menten curve in black. GraphPad Prism 8.0 analysis rendered the observed constants as K_M (obs): 28±2 μM, k_{cat} (obs): 56±3 s^{-1}. k_{cat} values were determined by dividing V_{max} (61 μM min^{-1}) by the total enzyme concentration (20 nM). Experiments were performed with 3 independent biological replicates, each of which had 3 technical replicates.

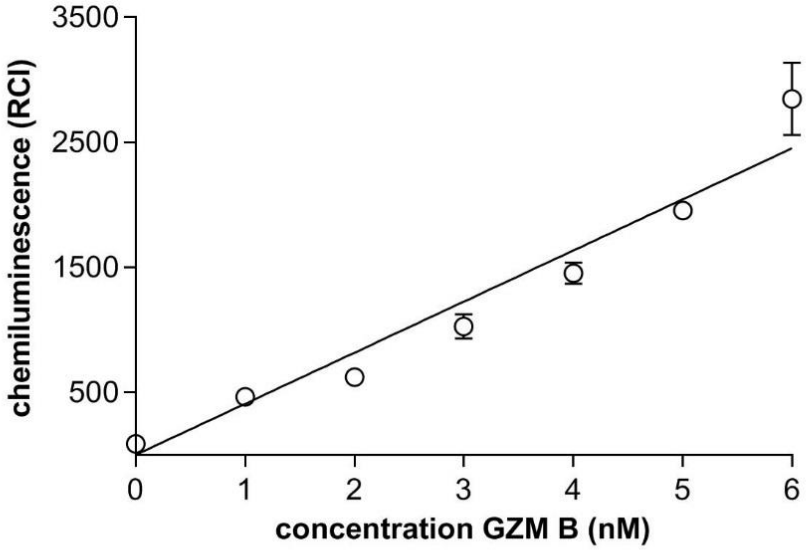

Supplementary Figure 3. Limit of detection of GZM B using probe 1. Determination of the limit of detection of human GZM B by chemiluminescence emission (528±20 nm) of probe **1** (100 μM) after reaction with increasing amounts of enzyme (1, 2, 3, 4, 5, 6 nM) at 37 ˚C for 10 min. Data presented as mean values ± SD (n≥3).

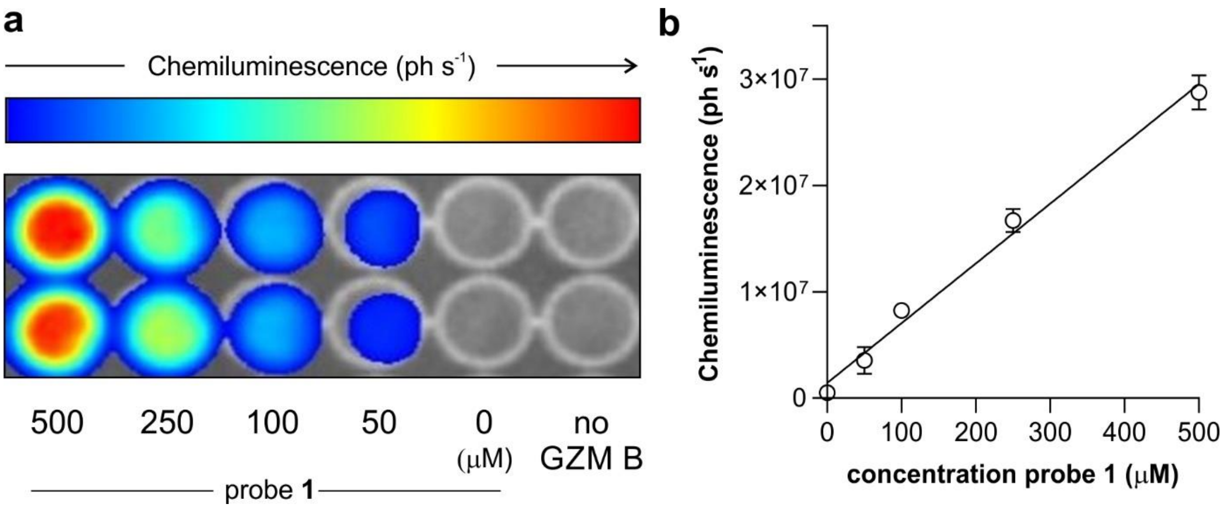

Supplementary Figure 4. **Detection of chemiluminescence activation of probe 1 with human GZM B under the PhotonImager™ imaging system.** a) Representative images of chemiluminescence emission of probe **1** (at the indicated concentrations) after addition to human GZM B (20 nM) in buffer containing 50 mM Tris-HCl and 100 mM NaCl (pH 7.4). Images were acquired immediately after addition of the probe **1** under a PhotonImager™ system and processed with M3Vision™ software. b) Quantification of chemiluminescence emission (ph s^{-1}) generated by probe **1** for each of the conditions displayed. Data presented as means ± SD (n=3).

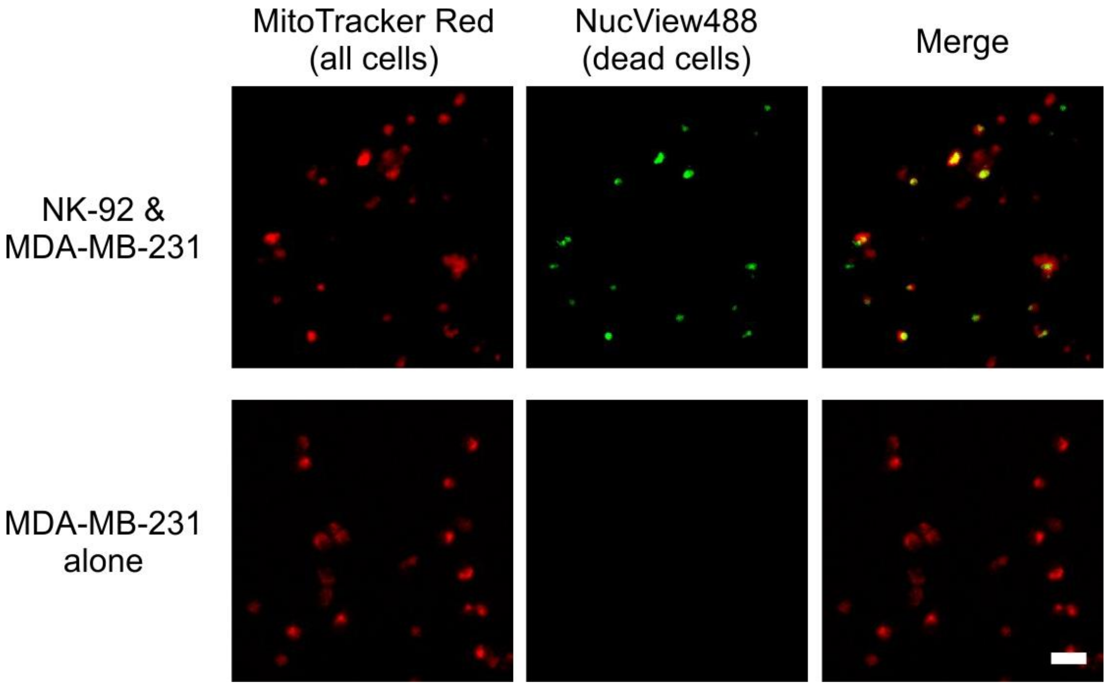

Supplementary Figure 5. **Detection of apoptosis in MDA-MB-231 cells in co-culture with NK-92 cells.** MDA-MB-231 cells were stained with MitoTracker Red CMXRos (300 nM, red) and incubated for 24 h with or without NK-92 cells at 37°C. Cells were then treated with NucView488 (10 μM, green) to stain apoptotic cells and imaged under the microscope. Fluorescence microscopy images were acquired in an EVOS FL Auto 2 widefield imaging system. Scale bar: 50 μm.

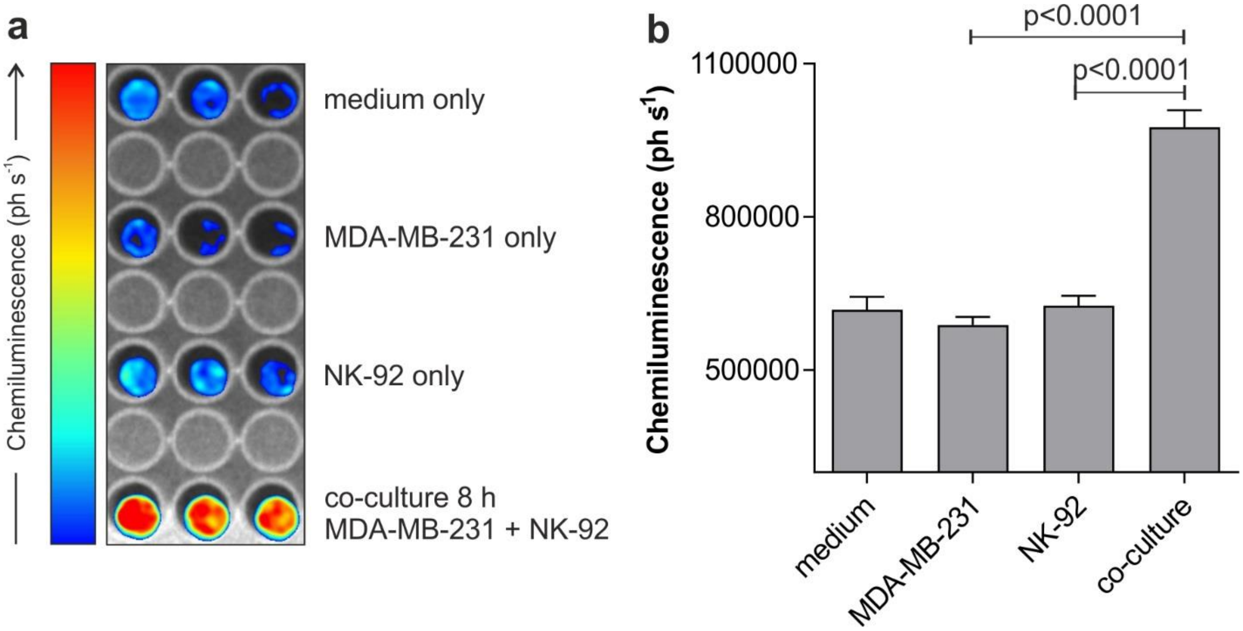

Supplementary Figure 6. **Detection of bright chemiluminescence from probe 1 in NK-92 cell/MDA-MB-231 co-cultures but not in individual cells.** a) Chemiluminescence images of probe **1** (100 µM) incubated with either medium only, MDA-MB-231 cells alone, NK-92 cells alone, or co-cultures of MDA-MB-231 and NK-92 cells (8:1 ratio). Images were acquired immediately after addition of the probe **1** under a PhotonImager™ system and processed with M3Vision™ software. b) Quantification of chemiluminescence emission (ph s^{-1}) generated by probe **1** for each of the conditions displayed. Data presented as mean values ± SEM (n=3). P values were obtained from ONE-ANOVA tests with multiple comparisons.

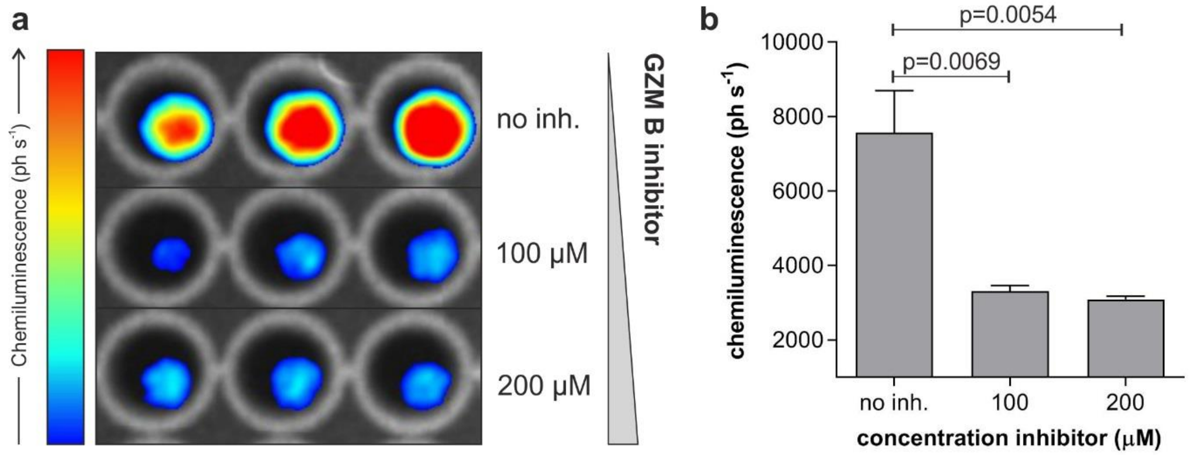

Supplementary Figure 7. Chemiluminescence activation of probe 1 is blocked by the GZM B inhibitor Ac-IEPD-CHO. a) Chemiluminescence images of co-cultures of MDA-MB-231 cells and NK-92 cells after incubation or not with the GZM B inhibitor Ac-IEPD-CHO for 1 h at 37 °C prior to addition of probe **1** (500 µM). Images were acquired immediately after addition of the probe **1** under a PhotonImager™ system and processed with M3Vision™ software. b) Quantification of chemiluminescence emission (ph s^{-1}) presented as means ± SEM (n=3). P values were obtained from ONE-ANOVA tests with multiple comparisons.

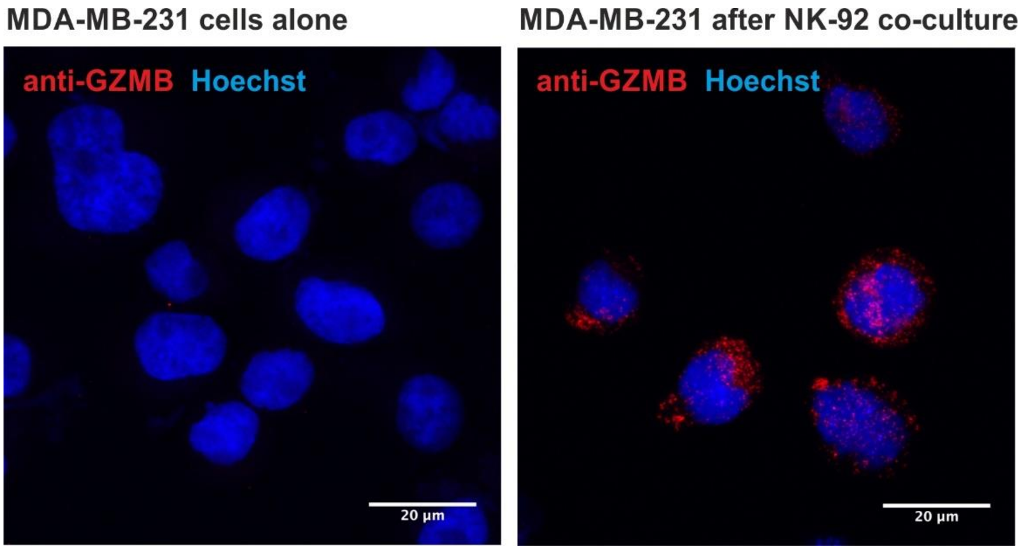

Supplementary Figure 8. **Imaging of intracellular GZM B levels in MDA-MB-231 cells before and after co-culture with NK-92 cells.** NK-92 and MDA-MB-231 cells were co-cultured at a 8:1 ratio in NK cell media including IL-2 (2,000 U mL^{-1}). MDA-MB-231 cells were cultured alone as a control. Cell were fixed with PFA and incubated with a rat monoclonal antibody against human GZM B and a secondary antibody Alexa Fluor 647 donkey anti-rat. Slides were incubated with Hoechst 33342 and images were acquired on a confocal fluorescence microscope Leica SP8 (λ_{exc}: 405 nm and 633 nm). Scale bar: 20 μm.

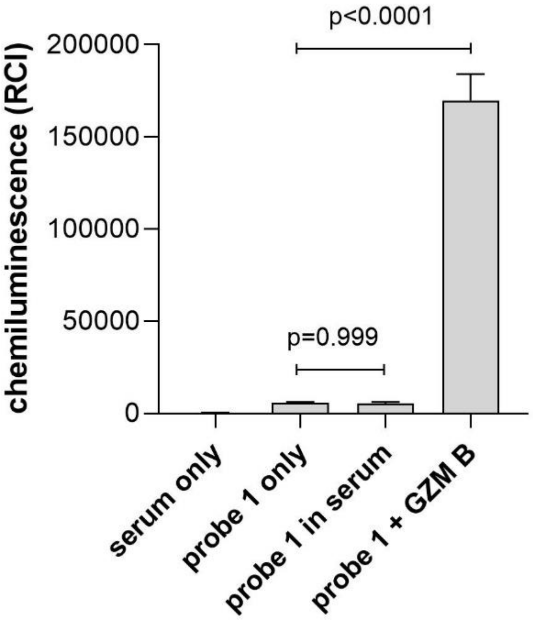

Supplementary Figure 9. Analysis of the stability of probe 1 in serum. Chemiluminescence emission of solutions containing serum alone, probe **1** (100 µM) alone, probe **1** (100 µM) incubated in serum for 1 h at 37° C, and probe **1** (100 µM) with GZM B (20 nM) for 1 h at 37° C. Data presented as means ± SEM (n=3). P values were obtained from ONE-ANOVA tests with multiple comparisons.

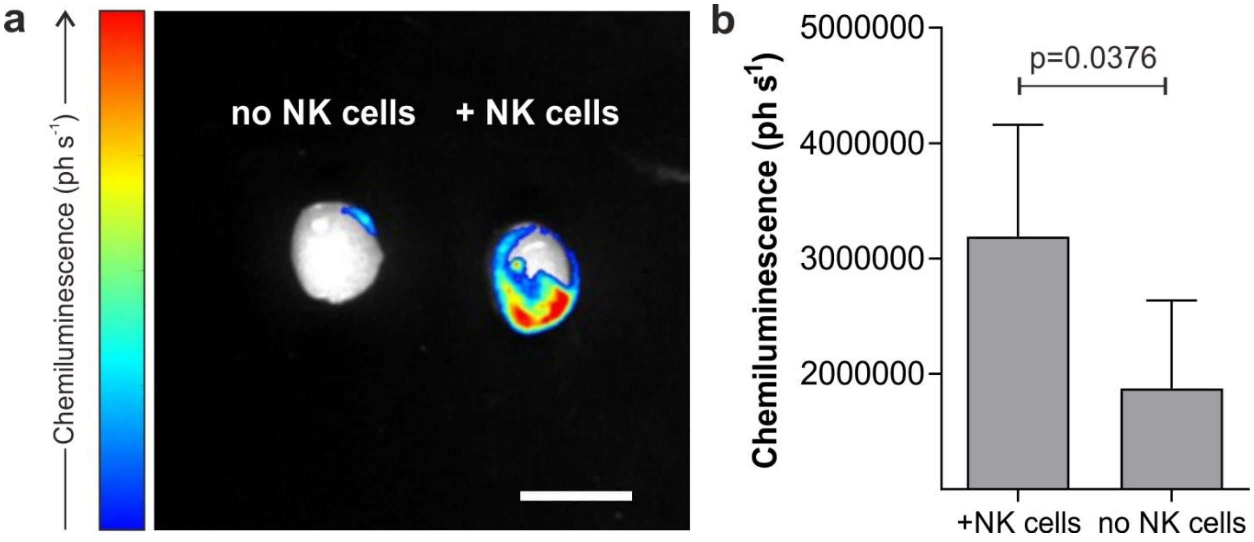

Supplementary Figure 10. Chemiluminescence activation of probe 1 detected *ex vivo* only in NK-cell containing tumours. a) Representative *ex vivo* chemiluminescence images of harvested MDA-MB-231 xenograft tumours (from 3 independent experiments) that had been injected with NK-92 cells (right) or not (left). Images were acquired under a PhotonImager system 5 min after incubation with probe **1** (100 μM) and processed with M3Vision™ software. Scale bar: 1 cm. b) Quantification of chemiluminescence emission (ph s^{-1}) presented as means ± SEM (n=3). P values were obtained from two-tailed paired *t* tests.

NMR spectra

^{1}H-NMR (compound **2**)

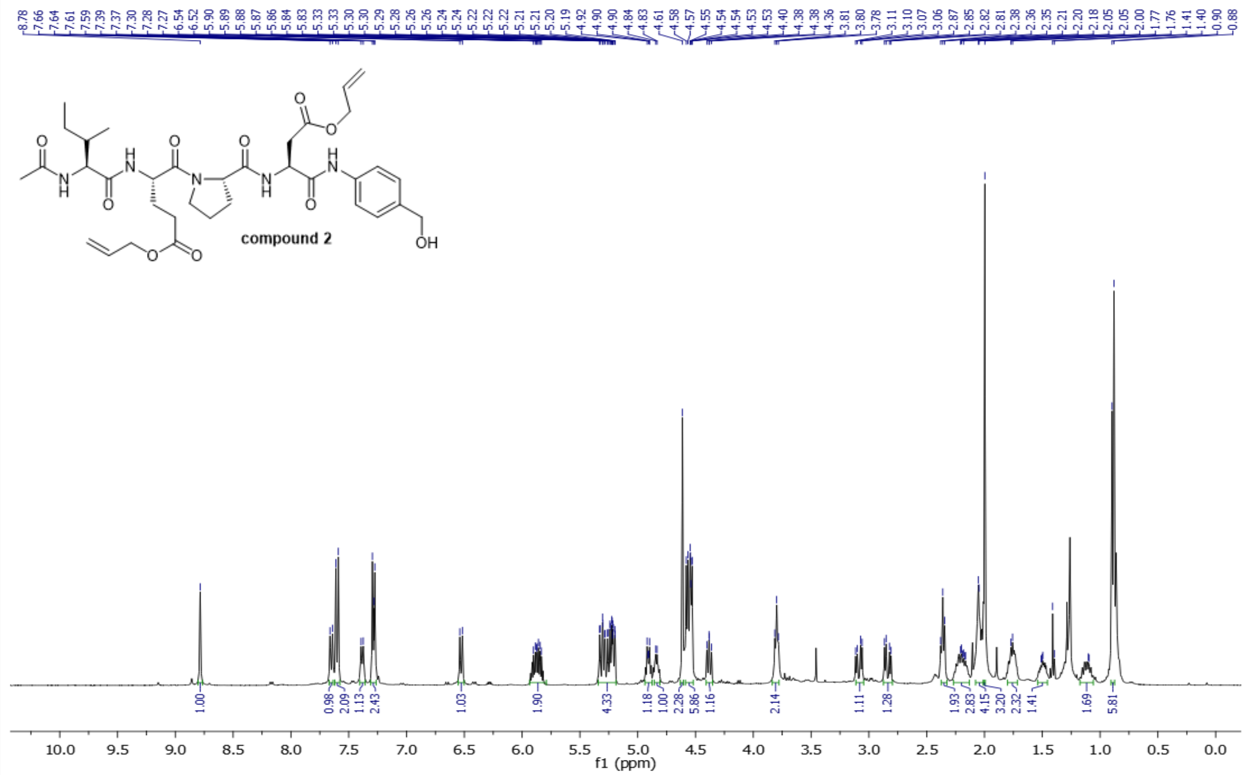

^{13}C-NMR (compound **2**)

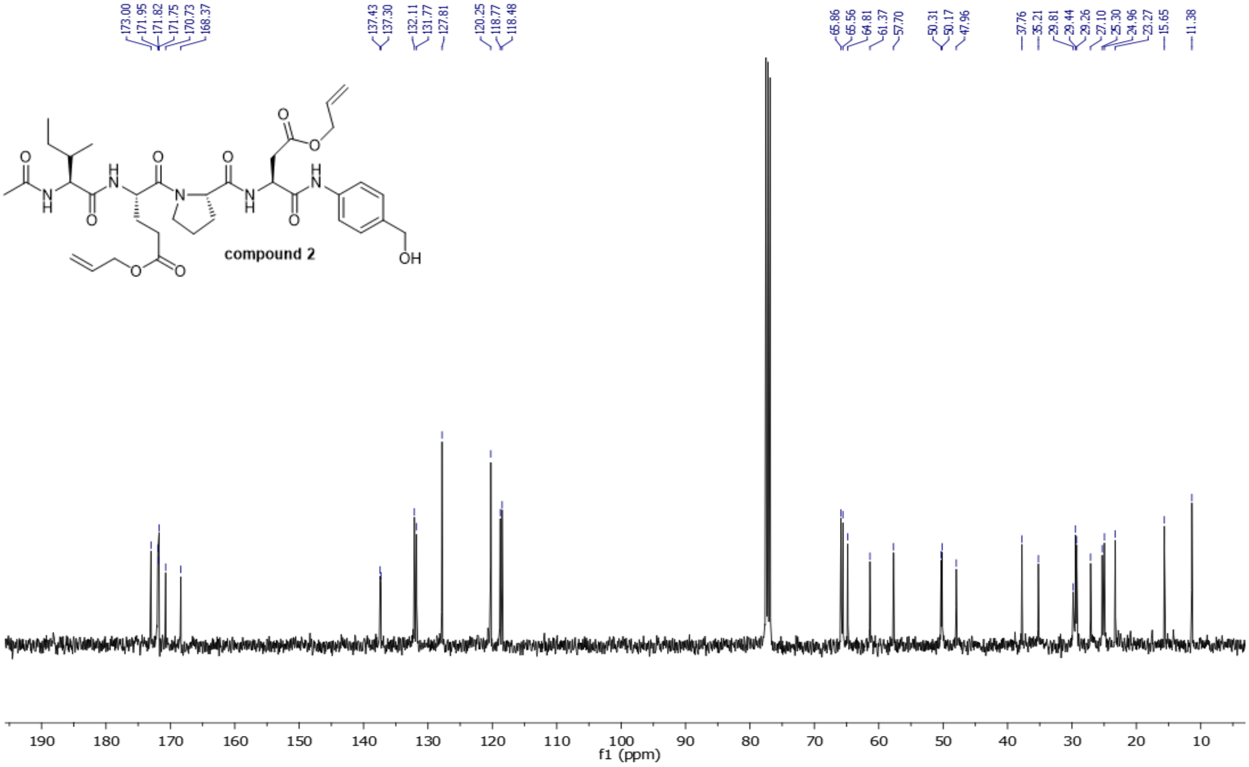

^{1}H-NMR (compound **3**)

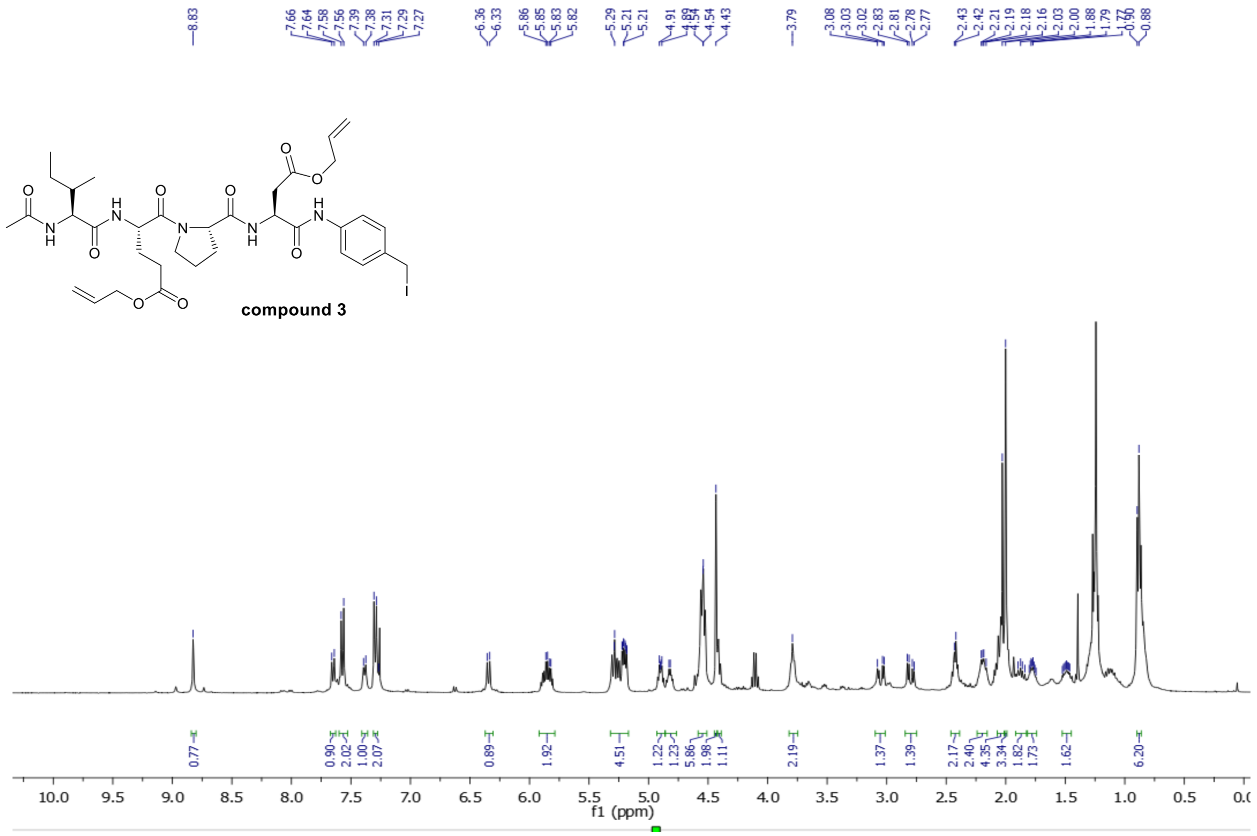

^{13}C-NMR (compound **3**)

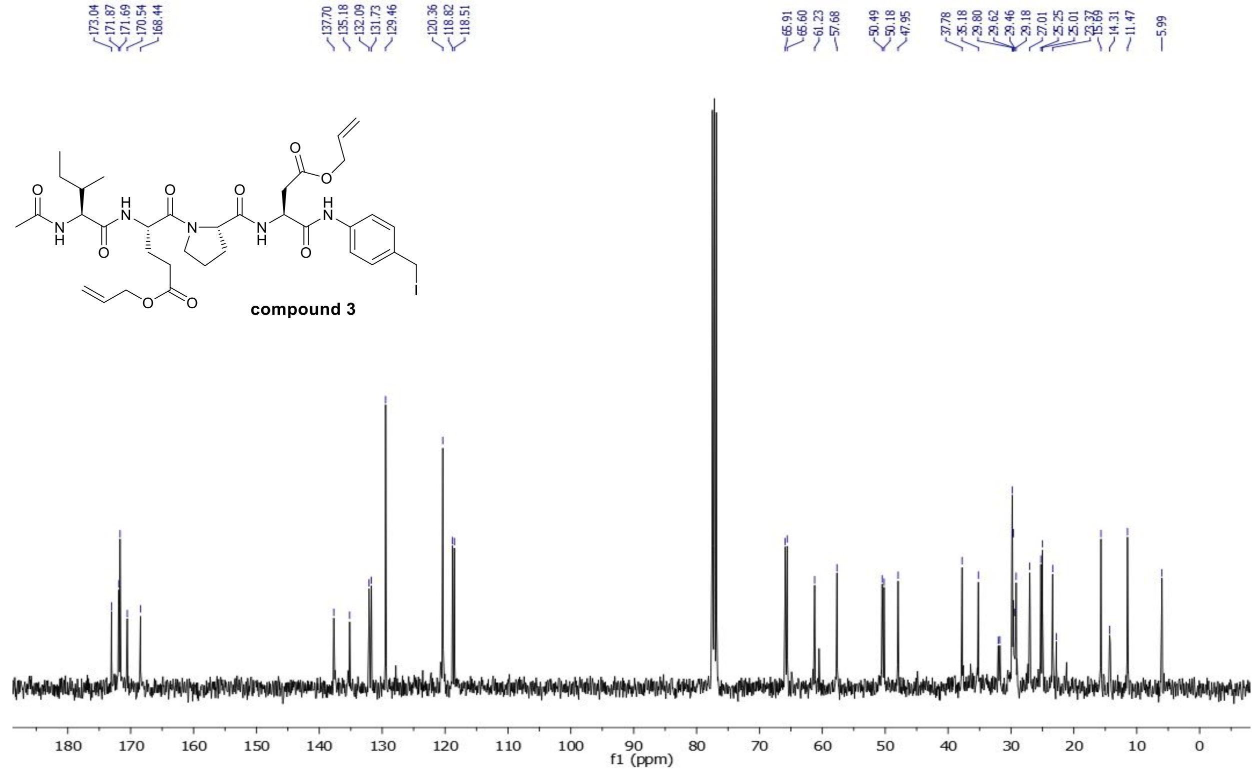

^{1}H-NMR (compound **4**)

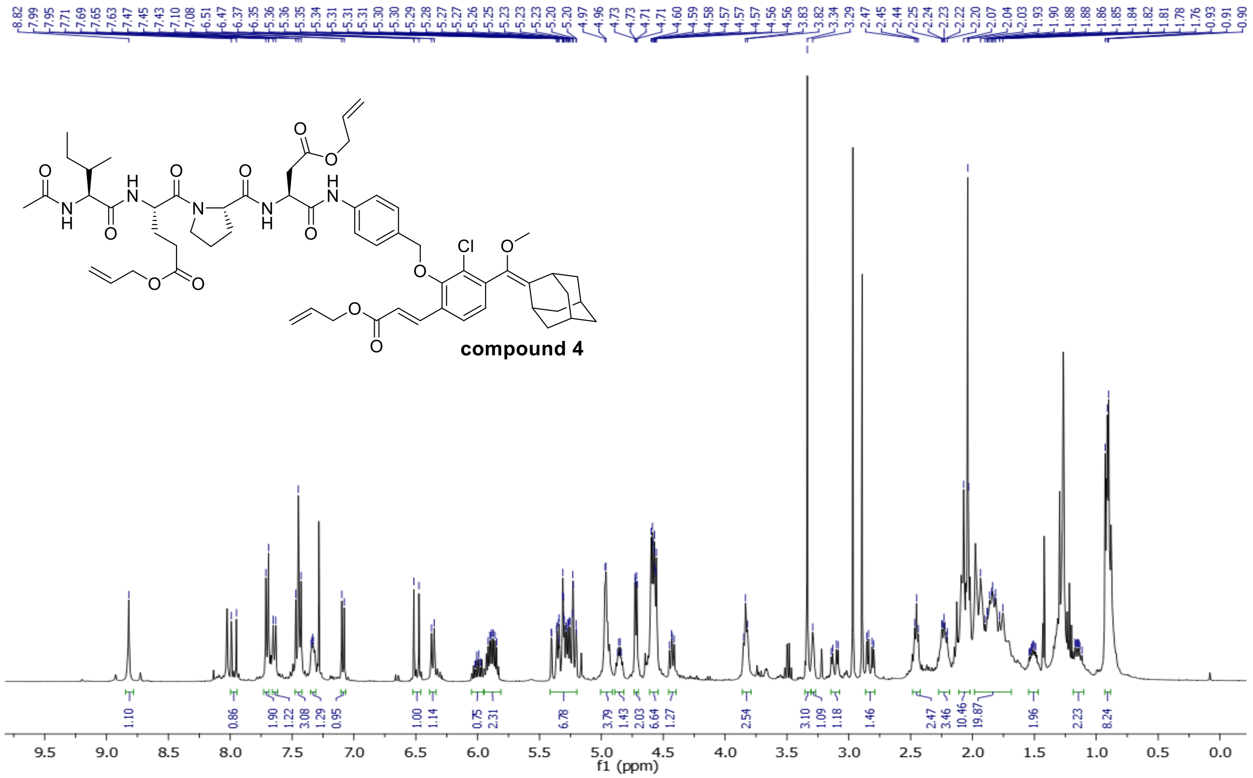

^{13}C-NMR (compound **4**)

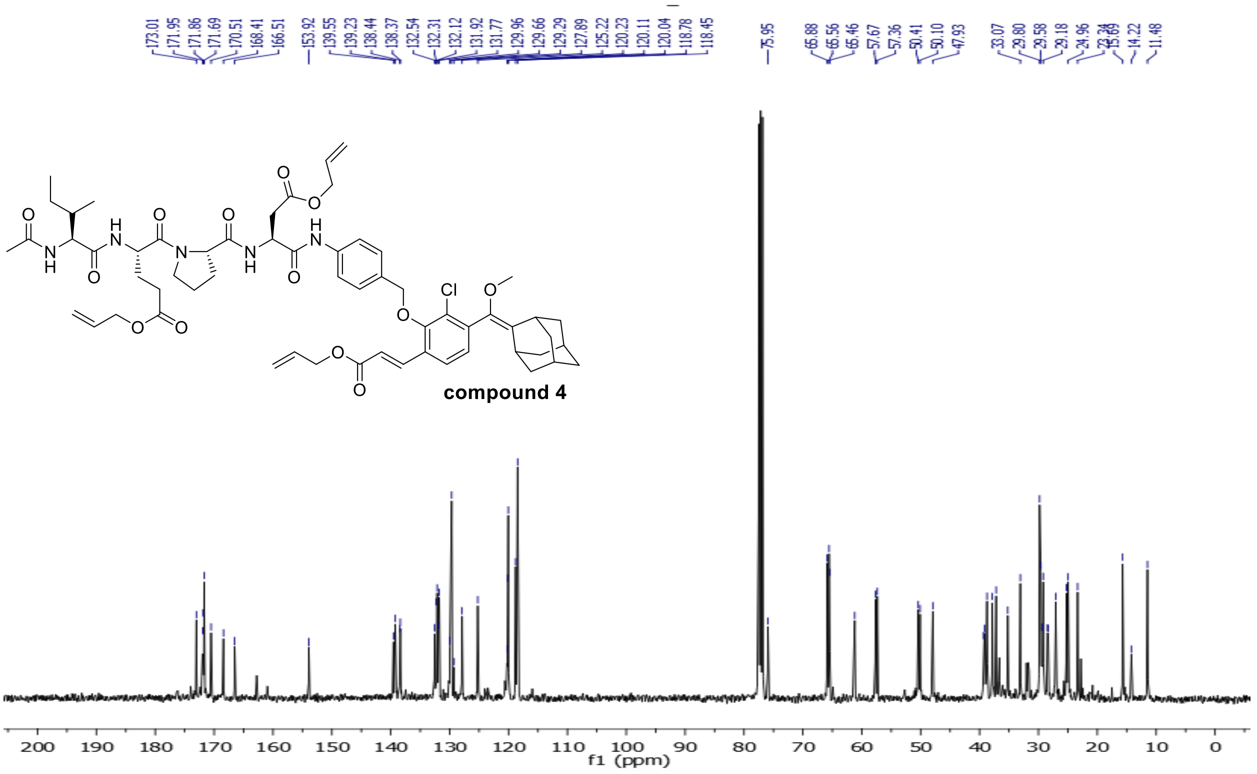

^{1}H-NMR (probe **1**)

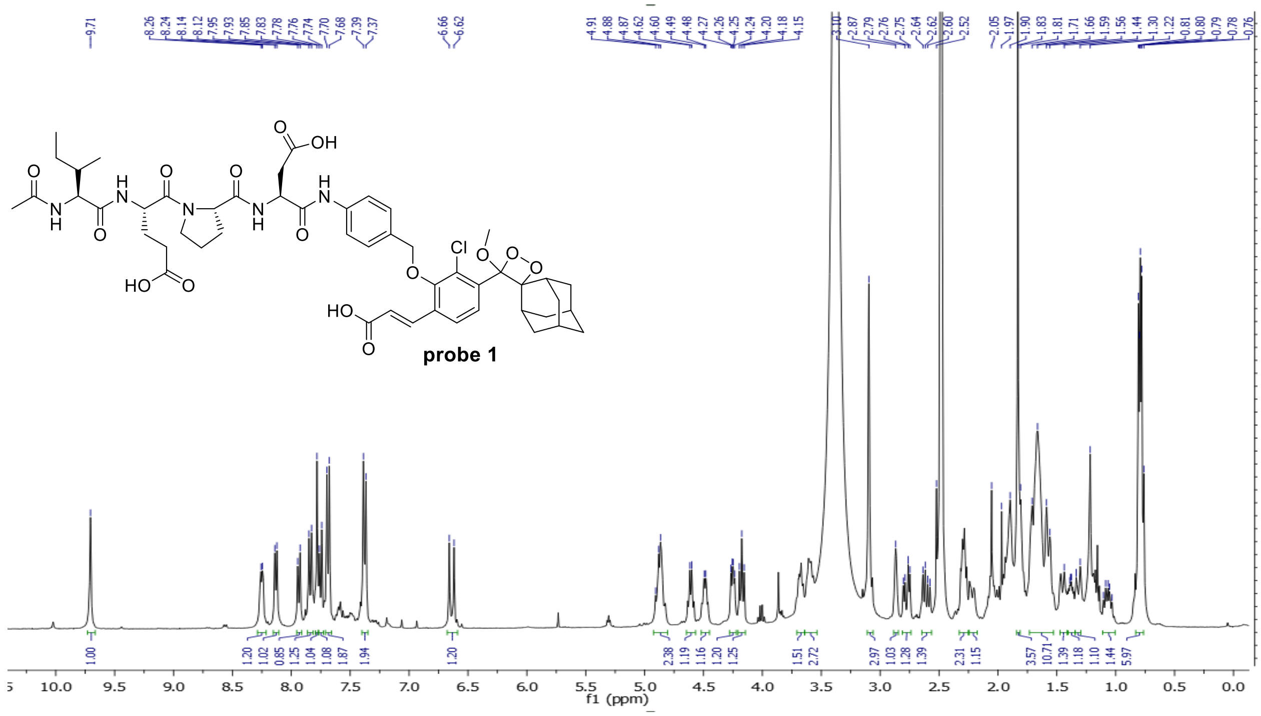

^{13}C-NMR (probe **1**)

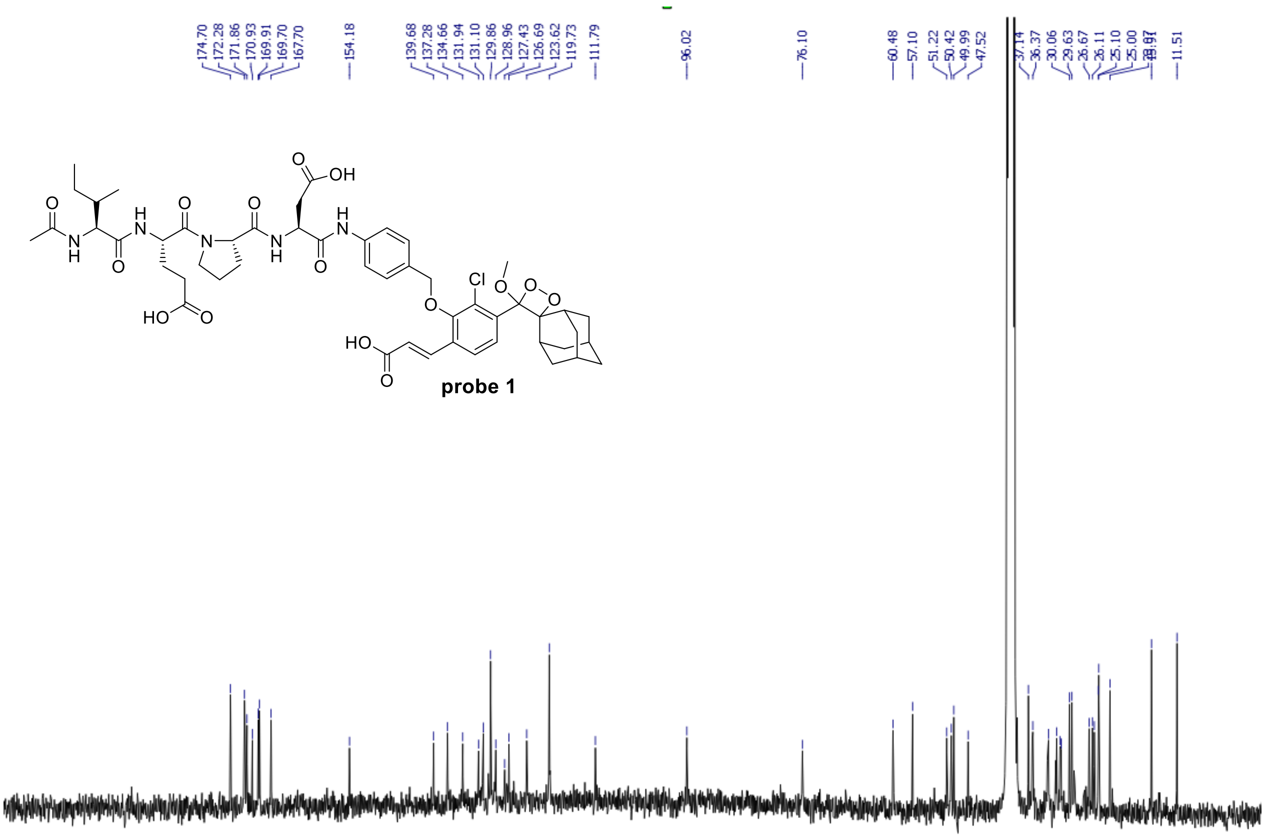

Supplementary references

1. Hananya, N., Reid, J.P., Green, O., Sigman, M.S., Shabat, D. Rapid chemiexcitation of dioxetane-based luminophores yields ultrasensitive chemiluminescence assays. *Chem. Sci.*, 2019, **10**, 1380-1385.
